# Surgeons’ Emotional Experience of Their Everyday Practice - A Qualitative Study

**DOI:** 10.1371/journal.pone.0143763

**Published:** 2015-11-24

**Authors:** Massimiliano Orri, Anne Revah-Lévy, Olivier Farges

**Affiliations:** 1 INSERM-U1178, 97 Boulevard de Port Royal, F-75679 Paris, France; 2 Université Paris-Sud, Université Paris-Descartes, Paris, France; 3 Department of hepatobiliopancreatic surgery and liver transplantation, Hôpital Beaujon, Assistance Publique Hôpitaux de Paris, F-92118 Clichy, France; 4 Centre de Soins Psychothérapeutiques de Transition pour Adolescents, Hôpital d'Argenteuil, F-75107 Argenteuil, France; 5 ECSTRA Team, UMR-1153, Inserm, Paris Diderot University, Sorbonne Paris Cité, Paris, France; 6 Université Paris Diderot, Sorbonne Paris Cité, Paris, France; University of Stirling, UNITED KINGDOM

## Abstract

**Background:**

Physicians’ emotions affect both patient care and personal well-being. Surgeons appear at particularly high risk, as evidenced by the high rate of burnout and the alarming consequences in both their personal lives and professional behavior. The aim of this qualitative study is to explore the emotional experiences of surgeons and their impact on their surgical practice.

**Methods and Findings:**

27 purposively selected liver and pancreatic surgeons from 10 teaching hospitals (23 men, 4 women) participated. Inclusion took place until data saturation was reached. Data were collected through individual interviews and thematically analyzed independently by 3 researchers (a psychologist, a psychiatrist, and a surgeon). 7 themes emerged from the analysis, categorized in 3 main or superordinate themes, which described surgeons’ emotional experience *before*, *during*, and *after surgery*. Burdensome emotions are present throughout all 3 periods (and invade life outside the hospital)—surgeons’ own emotions, their perception of patients’ emotions, and their entwinement. The interviewees described the range of emotional situations they face (with patients, families, colleagues), the influence of the institutional framework (time pressure and fatigue, cultural pressure to satisfy the ideal image of a surgeon), as well as the emotions they feel (including especially anxiety, fear, distress, guilt, and accountability).

**Conclusions:**

Emotions are ubiquitous in surgeons’ experience, and their exposure to stress is chronic rather than acute. Considering emotions only in terms of their relations to operative errors (as previous studies have done) is limiting. Although complications are quite rare events, the concern for possible complications is an oppressive experience, regardless of whether or not they actually occur.

## Introduction

The emotions physicians feel while caring for seriously ill patients affect both the doctors’ practices and their well-being, especially if they fail to examine these feelings [1]. Surgeons appear at particularly high risk, as evidenced both by a burnout rate that may be as high as 40% [2] and alarming consequences in both their personal lives (such as suicidal ideation, depression, alcohol consumption, and divorce) and professional behavior (errors, malpractice, disruptive behavior, early retirement) [2–5].

Data about surgeons’ emotions are sparse in the literature. The few available studies have mainly focused on those occurring in the context of stress, due either to an adverse event in the operating room [6] or the occurrence of a severe postoperative complication [7,8] and have explored their psychological repercussions on the so-called second victim [9]. These situations, although relevant, are potentially restrictive. Surgeons spend a limited portion of their working time in the operating room—less than 25% as senior surgeons [10], and less than 2 hours daily in the 80 working hours weekly as residents and junior surgeons [11]. Medical errors are relatively rare events, as shown by a recent study that found that 8.9% of surgeons surveyed reported concern that they had made a major medical error in the previous 3 months [12]. Because no study has extensively described the emotions surgeons feel in the different aspects of their everyday work, it remains unclear what role emotions play in surgical practice.

In a recent review exploring how surgeons described their practice, we showed a gap between the *myth of the surgeon* (idealized image of surgeons as non-introspective, isolated, cold technicians, derived from the culture of surgeons) and their actual experience [13]. This meta-synthesis showed that surgeons struggle to manage the emotions engendered by the patient-surgeon relationship. This exploratory study revealed an unexpected emotional feature of surgical practice, which called for an in-depth exploration

Starting from these findings, we designed a qualitative study aimed at describing the emotional aspects of surgeons’ daily practices. Our objectives were to describe the range of emotions that surgeons feel during their practice and the strategies they use to manage them.

## Materials and Methods

Because our objective was to explore surgeons’ perspectives about emotions, their possible causes and the coping strategies, we chose to use the grounded theory approach as general framework [14].

### Participants and sampling

Interviews were performed between April and November, 2012. Participants were hepato-pancreatico-biliary (HPB) surgeons. HPB surgery is high risk, associated with a 90-day mortality rate of 3 to 5% and a severe morbidity rate of 25%, and applied mainly to treat malignancies. According to the Grounded Theory methodology, a theoretical purposive sampling technique using maximum-variation [15] was used (sampling as wide a range of perspectives as possible to capture the broadest set of information and experiences, guided by the emerging data); accordingly, we interviewed surgeons with different ages, experience, academic positions, and geographical locations of practice. We initially contacted 27 surgeons, and all agreed to participate. The final sample size was determined by data saturation (i.e., the point at which no new themes emerged from the interviews) [16], which occurred after 24 interviews.

### Data collection

Data were collected through face-to-face unstructured interviews, all conducted by one researcher (MO) in French. The initiating prompt was “Can you describe in detail your last two working days—from the moment you woke up to the end of the day?” This approach suited our objective of obtaining an in-depth narrative; as the surgeons recalled their activity, the interviewer frequently prompted them to expand on their feelings and thoughts. The surgeons accepted open discussion easily and never focused only on the past two days, telling also about past experience and their general point of view.

### Data analysis

Each interview was transcribed verbatim and analyzed in French using thematic analysis. The analysis followed the grounded theory technique [17] After reading and re-reading of each interview, we developed the emergent themes following a series of coding steps: first (*open coding*), initial coding were generated by coding chunks of transcripts, keeping close to the participants’ words to isolate the basic units of meaning. Next (*axial coding*), we identified relations between the initial codes and grouped them into categories according to their similarity. Lastly (*selective coding*), these categories were organized into themes and subthemes. This inductive process (starting from the observation rather than from preexisting theories) was performed independently by 3 researchers with different backgrounds (MO, a psychologist; ARL, a psychiatrist; and OF, a surgeon) so that we could triangulate our different perspectives and attend systematically to our own effects as researchers at each step of the process [18]. Consensus was reached during study meetings. We used *NVIVO* software (QSR International, Melbourne, Australia) during the process of analysis.

Both the Health Information Protection Committee (CCTIRS) and the Data Protection Authority (CNIL) approved the study, and the surgeons interviewed provided their written consent. The reporting follows the COREQ statement [19].

## Results

Participants worked at 10 different teaching hospitals throughout France, their mean age was 45 years, and 4 were women; 14 were professors of surgery (including 7 department heads), 6 were qualified surgeons with a full-time contract, 5 were chief residents in surgery (6 to 9 years in training), and 2 surgical residents (second and fourth years in residency). The median interview lasted 48 minutes (range: 23–74 min).

Data analysis identified 7 main (superordinate) themes. They focused on the situations perceived by the surgeons to have significant emotional features. We organized these themes along the timeline of surgical care: *emotions before*, *during and after surgery*.

For each theme we propose 3 elements, corresponding to what surgeons described as their principal emotions, the possible causes, and their coping strategies (Table 1). The themes are described in detail in the next section, and excerpts of the participant discourse are reported to support our findings (see also S1 Table 1–3). Quotes are close translations that preserve original meaning rather than represent verbatim translations (i.e. they retained the feel of spoken rather than written language).

**Table 1:** Description of the main themes and subthemes.

Chronological description	Descriptive themes	Main elements (subthemes)	
Emotions Before Surgery	Preoperative Consultation as a Source of Emotion	Emotions	Responsibility of decision making
			Uncertainty surrounding this decision
			Taking a risk
		Causes	Subjectivity of clinical decisions
			Personal decisions prevail over team decisions
		Coping	Working in a team (but team decision not always applied)
			Shaping information and establishing an implicit contract
			Dealing with patients’ emotion
	Awaiting Surgery: Surgeons’ Anticipation of Difficulty	Emotions	Anxiety and fear
		Causes	Ubiquitous presence of the possibility of complication
		Coping	Mastering preoperative anxiety
Emotions During Surgery	Attempting to master their emotions through distancing and focusing on surgery as a technical activity	Emotions	Pleasure of operating, in a pleasant atmosphere
			Emotional identification with the patient
		Causes	Surgery as an aggressive act
			Operating on an individual human being
		Coping	Finding a balance between emotional involvement and neutralityThinking of surgery as a technical activity
		
	Occurrence of a complication	Emotions	Distress caused by the occurrence of a complication
			Anxiety due to losing a clear state of mind
		Causes	Occurrence of an intraoperative complicationFeeling responsible for this complication
		
		Coping	Cognitive re-centeringMinimizing evidence
		
	Distress caused by problems of time management and fatigue	Emotions	Pressure to be recognized as a good surgeon by the others
			Discomfort because of time pressure and fatigue
		Causes	No clear separation between work life and private life
			Perceived role expectations
		Coping	Acceptance of fatigue and satisfying the ideal surgeon image
			Further increase their work load and multitask
Emotions After Surgery	Repercussion of a complication	Emotions	Long-term burden of a complication
			Feeling of personal guilt and accountability
		Causes	Osmotic link between surgeon and patient
			Strengthened emotional link when complication occurs
	Pressures of the surgical ideal		Failure of case/facts to accord with the ideal position that of “surgery is the only chance for cure”
			Lack of a culture of non-accusatory error management
		Coping	“Image” of the surgeon; playing a roleMorbidity and Mortality meetings but their rationale (blame-free) not applied

### Emotions Before Surgery

#### Preoperative Consultation as a Source of Emotion

For surgeons, the preoperative consultation is the time when they decide whether or not to operate and make a recommendation to the patient. It necessitates taking personal responsibility for this decision, but also for the patient’s choice, because of the threat of potential complications.

The distinction between operations that can be done and those that should be done was not always clear, and the patient-surgeon relationship was sometimes the only criterion for decision:

I've got colleagues who tell me sometimes that NO, this patient should not be operated on! Because… blah blah blah… But in this situation, yes! I think he can! And the patient also and in our relationship, we think he can

(Surgeon_12)

Informing patients about the risks of the operation constitutes the core of the preoperative consultation. It is also its most emotional charged moment, as surgeons communicate information about the risks of death and of potentially major postoperative consequences. For this reason, they try to balance the benefits and risks of the intervention with the patient, or to put things into perspective to help patients deal with their fears:

I always try to put these things in relative terms, saying, for example, in this sentence which is a little blunt: “Did you come here by car? You know that you could have a fatal accident. Did you envision dying before getting into the car? Even if only by accident?”

(Surgeon_13).

Uncertainty was described, especially when the case is dangerous and surgeons must push the limits of their ability to offer the patient a chance at survival. The surgeons evoked the subjectivity of surgical decisions (described as strongly shaped by personal experience, clinical intuition, personal practices, and self-confidence) to explain the causes of this uncertainty.

Being a doctor means making decisions that have margins of errors or margins of risks that exist…

(Surgeon_07).

You have to take a risk. And the better you are, the more risks you take. So you have to accept that at some point! Damn but that's very hard!… very hard! Me, me, me, I'm going to make it! And you don't

(Surgeon_08).

Although decisions are made in teams, this does not reduce the surgeons’ uncertainty. They explicitly stated that the decisions made within their relationship with the patient overruled team decisions.

So, that’s what's perverse … these meetings must not make the decisions that are the individual doctor's responsibility… It's a unique relationship. You can get advice or consult your colleagues to check some things and ask their opinion, which can be useful, but there’s no way they can make the final decision…

(Surgeon_03).

One aim of preoperative consultation is therefore to establish an implicit contract of trust, intended to deal with the uncertainty and frame it. It is difficult to perform surgery outside that (both for the patient and the surgeon):

The patient has to tell himself that he is making me do it, that's it. He's making me do a complicated operation, which he can die from; for him to trust me he needs to see me as skilled, that's all. Therefore the patient gives us these skills […] that's what’s complicated, and you need experience to accept that you're not totally expert and that the patient trusts you anyway

(Surgeon_15).

The surgeons, recognizing that the preoperative consultation is emotionally charged for the patient, pointed out the need to deal with patients’ emotions during the information process. They stated that, ideally, all complications are supposed to be mentioned, but in practice, each surgeon has his or her own (implicit) criteria for choosing which complication to mention and how to discuss it. All seek to soften the emotional impact of the information delivered (S2 Table).

#### Awaiting Surgery: Surgeons’ Anticipation of Difficulty

These doctors described the possibility of intraoperative difficulties as a constant preoccupation before significant procedures. This concern has positive effects because it leads them to carefully analyze the patient's anatomy and various objective test results in order to predict the complexity of surgery. Alongside this preparation, however, the concern also results in worry when they anticipate the procedure will be difficult. Sometimes they even experience the approaching operation as a threat, when they are unsure that it will go as simply as planned or even that it will succeed.

These feelings needed to be mastered to maintain the relationship of trust established with the patient.

I can't let myself transmit my anxiety to the patient. I tell him that this surgery has a risk, but I can't say to him, for example, oh dear, I'm very anxious about operating on you, because then, he won’t come to the OR, not if he doesn't trust me… I need to transform my anxiety into an act that’s as safe as possible. So I take the maximum number of precautions so that the procedure will be done as safely as possible

(Surgeon_13).

They do not, however, always succeed in convincing themselves, as these worries occasionally invade their private life:

I went to bed, and it took, I think that it took me a solid hour to fall asleep… a solid hour… because I was thinking about things, about today's operation, which I was worrying about. It’s a really complicated procedure today… So … I knew that I was going to have a fairly complicated operation……

(Surgeon_12)

In some situations, tension is still higher, as when prophylactic surgery must be performed:

“preventive surgery, its basic requirement is zero complications, there must be no complications”

(Surgeon_12).

### Emotions During Surgery

Most of the time surgeons perform routine, ordinary tasks in the operating room (OR), in a pleasant atmosphere (where they chat with colleagues, teach young surgeons, and listen to music). Three sources of emotions were identified.

#### Attempting to master their emotions through distancing and focusing on surgery as a technical activity

Work in the OR was described by surgeons interviewed as a technical activity that provides scientific interest and personal pleasure:

… when we decide to do a procedure, we do it, and that’s the magic part, and it's very gratifying for us. We're artisans, we like our work, we are handymen, carpenters, masons, I don't know. We work on a material that's amusing, let's say, human material. But we could be woodworkers and make a chair that holds up, it's the same thing. But the patient doesn't know all that. He thinks we're taking care of him (laughs) but actually we're enjoying ourselves, more than anything

(Surgeon_15).

Moreover, an attitude detached from any relationship with the patient (i.e., seeing surgery only as a technical activity) is essential for many surgeons for the proper performance of the operation. Too much involvement (and perception of the patient as a human being, an individual) increases their risk of being “less scientific” and doing “shitty work”.

That keeps us from losing it in the OR and not daring to do anything. If you think that if you cut that, he might die, that if you do that, he might do this, […] if you get too involved emotionally and you decide you’re operating on Mrs X who has children, who loves flowers, uh, … on the actual day, you operate on a gall bladder

(Surgeon_06).

However, operating on a patient is charged with intense emotions, for two principal reasons. First, the very act of operating on someone establishes a strong emotional bond, one that grows stronger as the complexity of the case increases:

… in any case, the fact of operating on someone automatically creates a very strong bond… the fact of being authorized to open, for us in particular, someone else's gut, creates a closeness, a contact, a physical experience, and so a very particular responsibility

(Surgeon_09).

The second reason is that surgery is an aggressive act, potentially dangerous or lethal:

Surgery is an assault, don't you agree? It's an assault, opening a gut, it's assaultive, even if this opening is through tiny little holes of 1 cm, it's true. Surgery is hands that penetrate a body to repair or remove something, that's what surgery is

(Surgeon_01).

It’s each surgeon’s capacity to handle it, more or less well, because it's a job that's difficult, overall, I mean, not just physically! It's a difficult job… psychologically, because it's… you intervene… oh, I mean, it's… very invasive

(Surgeon_03).

Finding a balance between pleasure and threat, between emotional involvement and neutrality, is never straightforward, especially when the relationship with the patient is strong or the surgeon identifies with a patient.

When you are a little too emotionally involved in a situation, or with a patient, you can tend to worry too much, or, on the contrary, to minimize, not want to accept the complication and therefore not react, you have to be very very careful…

(Surgeon_03).

#### Occurrence of Complications

Intraoperative complications are among the most distressing events that can occur in the OR. Surgeons described fear and panic and subsequent wondering about potential catastrophically outcomes:

There was a moment… ten-fifteen minutes during which it was tough! You know […] So, that was really stressful…I was thinking, “shit! Will I bring it off? Yes, I will bring it off, but maybe I actually won’t!” I’m still asking this question! Even at my age! That’s rare eh, that’s rare for this to happen, but today it happened… “Shit, maybe today I won’t bring it off! If I fail in reconstructing this stuff….it will go very badly…” Fortunately I got it back! But my adrenal glands got wiped out!

(Surgeon_12).

In these moments, a clear mental condition, although difficult to maintain, is utterly essential for overcoming the stress. Sometimes emotions due to complications can overwhelm the surgeon’s state of mind, and a snowball effect can follow.

I did an arterial suture. I unclamped, and I saw it, and it was no good; so I redid the arterial suture, I unclamped, and it was still no good, …and I redid it and redid it…I redid it three times. I was in a kind of mental tunnel where I forgot everything else. My only objective was to finish the hepatic artery and for it to work well. That meant that I made an anastomosis once, twice, three times, and I forgot everything around me

(Surgeon_05).

#### Distress Caused by Problems of Time Management and Fatigue

Stressors also come from factors unrelated to either the ongoing operation or patient care. The first is time pressure and management, reported by both junior and senior surgeons—although for different reasons. Junior surgeons must demonstrate to their other co-workers (nurses, anesthetists, the team to follow them) that they are able to manage OR time effectively, to create a good reputation (“it’s how you start off that counts”).

Someone who operates fast, even if he operates badly, that's good, because he doesn't spend too much time, that's good for everyone

(Surgeon_06).

Senior surgeons can also be affected by external time constraints:

If I have two patients to do who are sort of complex and I have a meeting afterwards, at 5 in the afternoon or in the evening… I really need to put pressure on everyone to get a move on …

(Surgeon_14).

Fatigue is also a major distressing factor. It is perceived as an innate characteristic of surgery, generally enshrined in and sustained by surgical culture. Ability to deal with the erratic hours is a requirement for surgeons and a challenge for trainees.

We also have to be ready: first to accept a somewhat irregular lifestyle … tiring too… so we need to adapt to that… if you're not ready for that

(Surgeon_02).

There's a state of fatigue, when you have accumulated really lots and lots of patients, when you do lots of surgery, […] it is anxiety-inducing sometimes, to start saying, I'm tired; that's one of the things that are, that are, that can be very worrisome

(Surgeon_03).

### Emotions After Surgery

#### Repercussions of a complication

Complications negatively affect surgeons after they finish an operation. The related emotional burdens vary in their timing and manifestations (S3 Table), but were always described as horrendous:

How did I experience it? As a catastrophic failure, basically […] it was a really awful experience, the next day I was … It was horrible. Something I wouldn't wish on anyone, really, it was just horrible…

(Surgeon_12).

In some cases, surgeons reported struggling to acknowledge the occurrence of a complication and described their delayed awareness as denial:

…when the liver wasn’t good, when it wasn't working, I said, "this isn't possible, it's going to work, it will work, it will work" and it didn't ever work … because, in fact, for a while, I was blind to my mistake…

(Surgeon_05).

Every surgeon, regardless of experience or age, mentioned feeling responsible for postoperative complications, described as “feeling guilty”, and attributed this to the constitutionally interventional nature of surgery.

We are very invasive, necessarily at one moment or another, even if we step back … we are directly responsible for what we do, I mean, if it goes badly, a surgeon always feels guilty

(Surgeon_03)

During the postoperative period, surgeons’ emotions depend on their patients’ health status. Emotional involvement is the core concept that surgeons evoke to describe the strong entwinement with their patients (“When a patient isn’t well, the surgeon isn’t well, that’s certain…”). This is mirrored by their concrete involvement (eg, long working hours, remaining on call) and is the key feature according to the interviewees distinguishing surgeons from other physicians:

Surgeons have trouble leaving the hospital when their patient is doing badly. And they don’t take turns relieving each other, as other doctors do […] but the surgeon considers it is HIS patient because he did the operation and he is responsible for him [the patient] and what happens to him, and if it goes badly, it’s partly his fault. And that probably makes them more willing to be at the hospital from morning to evening, Saturday morning, Sunday, on call …

(Surgeon_4).

As surgeons clearly stated, the strength of their relationship with the patient (and his or her family) makes it even more difficult to deal with the patient’s complication or deterioration, from an emotional point of view:

That is something that is indeed very difficult in our job. That is, to cope with complications. Especially when you have built up a relationship, that is, something … a bond … that … oh … I'll say, … I don't know how to define it, but truly, the doctor-patient relationship that … that is made of trust. When people rely wholly on the doctor, and therefore, coping with complications, it's annoying… finally, still, you have to do it a little…

(Surgeon_03).

#### Pressures of the surgical ideal

Emotions due to postoperative complications are also exacerbated by the specific surgical cultural milieu, the myth of the surgeon. One issue is that many surgeons uphold the ideal that “surgery is the only chance for cure”, although many of the diseases they treat have very little chance of an actual cure. They thus described feelings of failure when surgery does not achieve this goal.

We're in a process where we operate on people, therefore we're in a process to cure them […] what we experience with a patient who has relapsed, who has metastases, it's practically a failure for us

(Surgeon_10).

Second, the institutional procedures and processes for discussing complications do not facilitate the integration of either complications or emotions into surgical culture. As one surgeon said: “We have a major problem in medicine and in surgery, that we have no ‘culture of errors’…” (Surgeon_05). Feelings are thus aggravated by the emotional impact of relations between OR team members. Surgeons described the pressure to meet the expectations of their surgical culture:

When you’re very young, when you're a resident, well, then it's rubbish, you do stupid things, that's what it is. At the beginning it's training, you are supposed to be trained. When you're no longer so young, you still do stupid things … but you can't do too many, because normally you need to have some expertise that justifies that you're where you are now. […] which is a phase where…where you still feel that your peers are evaluating you, which is an important phase. Because precisely, in this phase, you are not allowed to make mistakes. And when you are even older, you can do stupid things, because finally you have saved so many patients that you can do stupid things

(Surgeon_05).

Surgeons considered that the way in which institutions deal with examining complication were not closely related to the ostensible goal of improving patient care. Morbidity and mortality meetings, for example, are experienced as a way to find out who was responsible—even if in a socially accepted way.

Even if there was a desire to do precisely this … morbidity and mortality meetings to discuss all this, somewhere there is always some amount of … judgment …

(Surgeon_05).

Moreover, when surgeons informally discuss complications, mistakes, and poor outcomes with colleagues, implicit cultural rules impose a way of discussing them that is ineffective in appeasing these emotions.

They told me, no you couldn't do it a different way, blah blah, to please me, I think […] I'm the old man on the team, so the guys, my collaborators, I think they like me a lot and that they,…they didn't lay into me…

(Surgeon_12).

## Discussion

Previous investigations of surgeons’ emotions have focused mainly on the acute context of stress due to either an adverse event in the operating room [6] or the occurrence of a severe postoperative complication [7]. The present study shows that addressing only these acute situations, although they are relevant, is inadequate. Surgeons’ emotions (including anxiety, fear, distress, guilt, and accountability) occur even in the absence of an adverse event and pervade surgeons’ relation with patients, families or colleagues. Our study of the causes of these emotions and the coping strategies that surgeons use shows that they appear to be embedded in a structured cultural network and thus provides new insights into the practice of surgery and the malaise of the surgical profession. The analysis of HPB surgeons’ narratives we presented allowed us to generate some insights about high-risk surgical practice in general.

Indeed, the interviewees descriptions made clear that the emotional situations they face are present throughout the preoperative, intraoperative, and postoperative periods (and invade life outside the hospital). Consequently, surgeons’ exposure to stress is not an acute but a chronic experience. This observation is important as psychoneuroimmunology studies have shown the predominant role of chronic, compared to acute, stressors in the onset of both somatic and psychological diseases [20,21]. Moreover, the surgeons did not distinguish acute from chronic stressors. This is evident, for example, in the narratives about complications in which surgeons failed to differentiate between *actual* complications, acute stressors that are fortunately rare [12], and *potential* complications, chronic stressors that overwhelm their daily practice and the pre- intra- and postoperative relationships they have with their patients. Surgery is therefore experienced simultaneously as an act that might save and an act that might kill [a patient, an organ, a function]. This paradox (which makes the *threat* of the complication omnipresent in surgeons’ lives) is unique to surgical specialties and probably has no equivalent in other professions.

The three root causes of emotions identified in surgeons’ narratives relate to the practice of surgery, rather than to the actual occurrence of an adverse event. First, interviewees described surgery as an aggressive act performed on a human being and the unique bond which it creates between them and their patients. This bond has been shown in this and previous studies to be particularly tight when surgeons identify with the patient or when the case is challenging [13]. Second, they acknowledged their subjectivity and uncertainty in every aspect of their practice. Surgical decision making is indeed a subjective balance between competing avowed, unavowed and disavowed priorities [22]. The uncertainty regarding surgeons’ decisions translates into inaccurate predictions of the outcome of surgery [23,24]. Third, surgeons considered the institutional framework in which they work a source of pressure or fatigue and more generally as being unsupportive [7]. These root causes reinforce surgeons’ feeling of loneliness and the gap between their perceived need to satisfy the cultural image of surgeons as strong, decisive and responsible for their patients [25,26] and the reality of their daily work [13].

Surgeons in this study mentioned several coping strategies that have proven to be effective, including teamwork [27,28], recognizing patients’ emotions [29], balancing personal involvement and neutrality [30], and cognitive re-centering [31]. They also described, however, how these strategies may be used ineffectively or diverted from their rationale. In the preoperative period, surgeons’ personal (subjective) decisions ultimately prevail over group decisions. Moreover, these personal decisions are made as part of an implicit contract between the surgeon and the patient, a contract that has been shown to be fragile and to have negative effects on patient-centered care [32]. During the operation, surgeons keep other team members distant at emotionally charged moments and rarely mention them in their narratives. This observation parallels the difference in the perceived role of, and the definition of performance by, surgeons, anesthetists, and scrub nurses [33]. The OR is also the place where disruptive behaviors typically occur [5]. In the postoperative period, surgeons cannot refrain from thinking about patient morbidity and mortality meetings in terms of their own *individual issues* (surgeons’ individual responsibility) rather than in terms of *patient* or *system issues*. In other medical fields, emotions are managed differently at an institutional level: physicians acknowledge these emotions and discuss them in group or supervision sessions (for instance, during “Balint groups” [34,35] in oncology departments). Emotions are even used as a therapeutic tool [36–38]. The reasons for the ineffectiveness of surgeons’ coping strategies are therefore related to both the surgical culture of individualism [25,26], and the institutional organization, which does not support surgeons.

This gap between surgeons as individuals and the environment they work in strengthens their feelings of loneliness, an emotion made obvious throughout the interviews, where they restricted surgery to a technical act and found their only escape in *distancing through technical prowess*. This coping strategy bears the inherent risk (explicitly described by the interviewees) of considering surgery as *“the only chance for cure”*, *“pushing the limits [of surgical indication]” as the way to “offer [their patients] a chance”*, and considering that *“what is technically feasible should be performed”*. By doing so, surgeons cast themselves as the only active actor in what they perceive as a necessary but uncertain drama that may result in either life or death (success or failure).

The elements of surgeons’ emotions (their nature, their cause, the coping strategies used) therefore appear embedded in a structured cultural network that governs surgical practice. The interaction of these elements may explain the acknowledged malaise of the surgical profession. The continuous flood of emotions (recognized or not) paves the way for emotional exhaustion; distancing through technical activity is a way of disconnecting from or dehumanizing others and thus of depersonalization; performing surgery with subjectivity and casting oneself as the sole actor responsible for its outcome entails, among other things, an innate risk of personal failure. Emotional exhaustion, depersonalization, and a sense of low personal accomplishment are the three symptoms of burnout [39].

The main strength of this study is the specific qualitative approach used. Instead of questioning surgeons about particular emotional issues by specific prompts, as most previous studies have done [7,8] with the built-in risk of promoting technical answers, we chose a simple technical question (i.e., describe an average working day) to start the interview. Interviews were performed face-to-face by a psychologist who was not known by the surgeons interviewed, and thematic analysis of the interviews was performed by three researchers, each with a different background. The interviewees were purposively sampled by gender, academic position, experience, and workplace to include both information-rich participants (in particular the most senior surgeons) and informants who would potentially contradict or seem to contradict the findings (in particular, the most junior surgeons), according to Grounded Theory theoretical sampling procedure.

There are also certain limitations that need to be considered. First, the qualitative approach used in this study allows hypotheses to be formulated but is not designed to confirm them. Second, our sample of surgeons performed HPB surgery, which is considered a high-risk specialty. In addition, these surgeons practice in a single country and in the specific setting of teaching hospitals. Assessing the transferability of our findings will therefore be interesting, for each specialty may have its own specificity, the health care system or the cultural attitudes towards surgeons may vary between countries and team-based sharing of responsibilities and accountability might be perceived differently from one department to another. It is however worth mentioning that none of the themes or subthemes in this study are specific to HPB surgery and that the prevalence of surgeons’ psychological morbidity does not differ between countries, specialties, age groups, surgical grades, genders or according to hours worked per week [40,41].

Because emotion is a subjective phenomenon and surgeons have been shown to calibrate their level of distress unreliably [42], they must be taught to understand that the way they describe their work contains the seeds of burnout. The first—difficult—step must be to achieve insight and acceptance of the emotional difficulty inherent in being a surgeon:

“A surgeon’s life is full of complications”

(Surgeon_4).

## Supporting Information

S1 Table Additional quotes from the interviews for each theme. (DOC)

S2 Table Strategies used by surgeons to manage the patients’ emotions during the information process. (DOC)

S3 Table Domains of emotional impairment. (DOC)
