# Supplementary material for: Surgeons’ Emotional Experience of Their Everyday Practice - A Qualitative Study
Source: PLoS One. 2015 Nov 24;10(11):e0143763. doi: 10.1371/journal.pone.0143763 (PMC4657990; doi:10.1371/journal.pone.0143763)
Supplement: S1 Table — (DOC) [file pone.0143763.s001.doc]

**A.** Quotes exemplifying emotions before surgery.

| **Preoperative Consultation as a Source of Emotion** |
| --- |
| **Preoperative contract: establishing a relationship of trust**  There's a very strong moral contract that links you to the patient. That is, you don't let go... (Surgeon_09).  He came trusting me […] they could see someone else but they came to see me, so I need to be irreproachable in what I do to them… … Because they trusted me and it's huge to put their life in my hands... so I owe it to myself to be irreproachable for that (Surgeon_06).  Sometimes you have to operate on someone else's patient, but that's totally different because, first, for me, I find psychologically, I find that I would rather operate on a patient I know because I've spoken to him, I've seen him, he's met me, there's a relationship of trust between us, there's trust…. (Resident_02). |
| **Subjectivity of surgical decisions**  … The elements are always somewhat the same, things about the doctor who sees the patient, how he is, so, his craft where he learned the signs, you know… after the operation he sees that there is a drain, two drains that worry him, a fever, etc… and then there are the elements linked to other tests you can order, and then, everything, all mixed up, in a kind of mixture, uh… since medicine is certainly not a science, these are going to lead to a decision or not, often according to the experience of the person who perceived all these signals… (Surgeon_02). |
| **Influence of the relationship on decision making**  So it's very interesting, precisely, the clinical practice guidelines I was talking to you about, that we talked about at the beginning… There's the guidelines and the actual patient, and fortunately, what we always say, the guidelines are for consulting ... they don't make the decisions! […] So that’s what's perverse … these meetings must not make the decisions that are the individual doctor's responsibility… It's a unique relationship. You can ask for advice or consult your colleagues to check some things and ask their opinions, but there’s no way they can make the final decision … […] I've got colleagues who tell me sometimes that NO, this patient should not be operated on! Because… blah blah blah… But in this situation, yes! I think he can! And the patient also and in our relationship, we think he can (Surgeon_03). |
| **Communication of risks**  And yes, they need to understand, they say, oh wow I'm afraid, I'm going to die with the operation, and I tell them that they have it backwards. If we do nothing, what are you going to do? You are going to die. So it is dangerous to do nothing, you must understand that doing nothing is much more dangerous than doing something. So they understand after; we need to straighten them out, right away (Surgeon_15). |
| **Awaiting Surgery: Surgeons’ Anticipation of Difficulty** |
| **Feelings of anxiety**  There are things that don't worry me anymore, operations that don't worry me any more at all, so I get there, I'm cool, I'm not at all anxious. Today I was a little, because I knew it was going to be difficult, I knew that it was risky, that the operation was complex, risky (Surgeon_12).  So, really, if you want, that was the … anxiety, because I think we are always a little anxious… The anxiety was whether I was going to succeed in extirpating the lesion as easily as that… Because my basic mission was much more limited, that there be no collateral damage, that the blood vessels be absolutely untouched, for me in any case… and I must not create problems where there hadn't been any before… And therefore it's ... the enigma was whether it was going to be simple or not… (Surgeon_13). |
| **Repercussions on personal life**  I went to bed and it took me, I think, an hour to fall asleep… a good hour … because I was thinking of things, about the operation today, which worried me, nonetheless, it was a really complicated operation today… So ...  Psychologist: Were you thinking about the operation the next day?»  Surgeon: Yeah, I thought about it a little… Because I knew I was going to have a pretty complicated operation… (Surgeon_12). |
| **Performing preventive surgery**  Contrary to what you might expect, removing a benign tumor is more anxiety-producing than removing a malignant tumors… yes… because taking out a malignant tumor, it's a patient… You have to remember that this patient didn't complain about anything, his tumor was discovered by chance. […]And therefore this preventive surgery, it has to have absolutely zero complications, you cannot allow any complications […]Therefore for this man, the pressure -- psychological, shall we say, it was only in the sense that there had to be zero intraoperative complications, and there must always be zero postoperative… (Surgeon_13). |

**B.** Quotes exemplifying emotions during surgery.

| **Distancing oneself from emotions by focusing on surgery as a technical activity** |
| --- |
| **Surgery as a scientific and technical activity**  When we decide to do a procedure, we do it, and that’s the magic part, and it's very gratifying for us. We're artisans, we like our work, we are handymen, carpenters, masons, I don't know. We work on a material that's amusing, let's say, human material. But we could be woodworkers and make a chair that holds up, it's the same thing. But the patient doesn't know all that. He thinks we're taking care of him (laughs) but actually, we're enjoying ourselves, more than anything. (Surgeon_15). |
| **Surgery as an aggressive act**  Surgery, it's an assault, don't you agree? It's an assault, open a gut, it's assaultive […] it's difficult for a patient … to accept this aggression even if he knows it's for his own good, if it's by someone he doesn't trust (Resident_01). |
| **Detachment as a requirement to perform surgery properly**  That keeps us from losing it in the OR and not daring to do anything. If you think that if you cut that, he might die, that if you do that, he might do this … […] if you get too involved emotionally and you decide you’re operating on Mrs X who has children, who loves flowers, but … on the actual day, you operate on a gall bladder (Surgeon_06).  You're not, I find that you're not in the right conditions for intraoperative decisions and motivation, doubt, ... and then there's a decision that's ... not easy to make about whether or not it's possible to resect the tumor, tumors like that which are pretty developed, so I've found that it's difficult. Anyway … !! (Surgeon_08). |
| **Operating on a patient creates a bond**  … in any case, the fact of operating on someone automatically creates a very strong bond. […] the fact of being authorized to open, in particular for us, someone else’s gut, creates a closeness, a contact, a physical experience, and so a very particular responsibility (Surgeon_09). |
| **Identifying with a patient**  Because after all that, we are human beings, and sometimes we make compassionate decisions. That is, if you operate on someone who, for example, is the same age as you or me, who has children the same age as me, and you find a large tumor of the pancreas, I would be more likely to say, … I'm going to force things a little, and I'm going to remove it rather than just reclose it. (Surgeon_10).  ...when you are a little too emotionally involved in a situation, or with a patient, you can tend to worry too much, or, on the contrary, to minimize, not want to accept the complication and therefore not react, you have to be very very careful… It's something that is in fact very difficult in our job. That is, coping with complications. Especially when you have developed a relationship, …a something … a bond...which... that’s what I think (Surgeon_03). |
| **Occurrence of a complication** |
| **Emotions when complication occurs**  There was a moment… ten-fifteen minutes during which it was tough! You know […] So, that was really stressful…I was thinking, “shit! Will I bring it off? Yes, I will bring it off, but maybe I actually won’t!” I’m still asking this question! Even at my age! That’s rare eh, that’s rare for this to happen, but today it happened… “Shit, maybe today I won’t bring it off! If I fail in reconstructing this stuff….it will go very badly…” Fortunately I got it back! But my adrenal glands got wiped out! (Surgeon_12). |
| **Struggle to keep a clear state of mind**  At that point, you have to try to not to redo something stupid on top of it, because otherwise it all starts to accumulate a little…[…] it's not a very comfortable situation but, ok, afterwards, at a given moment, you always need to think that you can't give up. So then you have to concentrate again, calm yourself, and succeed (Surgeon_10).  I did the arterial suture, I unclamped, and I looked at it, and it wasn't good; so I redid the suture, I unclamped, and it still wasn't good …and I redid it and I redid it…I redid it three times. I was in a kind of mental tunnel where I forgot everything else. My only objective was to finish the hepatic artery and for it to work well. That means I did it once, twice, three times, and I forgot everything else around (Surgeon_05). |
| **Distress caused by time management and fatigue problems** |
| **Time management**  For some of the staff, especially the non-surgeon personnel, the operative time is more important, because they don't see the patients in post-op, so for them operative time is a criterion of quality. Someone who operates fast, even if he operates poorly, is good, because he doesn't spend much time, that's convenient for everyone. So yes, it's true that the goal is to operate well, but it must not be too long either, so that the non-surgeon team and the anesthetists are happy and trust you for future operations. (Surgeon_06).  What stresses me out isn't that at all, it's the idea that after the operation, I have something to do and so I need to have finished by such and such a time because otherwise I'm going to miss my thing, and that after, after, after, ... that's all. And so already in the morning I'm under pressure because if my schedule is busy, which was not too much the case yesterday, if I have two patients a little complex to do, and if I have a meeting afterwards, at 5 o'clock in the afternoon or in the evening…. Then I really need to put pressure on everyone to get a move on … (Surgeon_14). |
| **Fatigue**  there's a state of fatigue, when you have acquired really lots and lots of patients, when you do lots of surgery, certainly, you acquire a fair amount of dexterity with experience but you also accumulate a fair amount of fatigue… so .[…] it is anxiety-inducing sometimes, to start saying, I'm tired; that's one of the things that are, that can be very worrisome (Surgeon_03).  We also have to be ready, first to accept a somewhat irregular lifestyle … tiring too… therefore we need to adapt to that… if you're not ready for that (Surgeon_02). |

**C.** Quotes exemplifying emotions after surgery

| **Repercussions of operative complications** |
| --- |
| **Facing complications**  That is something that is indeed very difficult in our job. That is, coping with complications. Especially when you've built up a relationship … that is, something … a bond ... that ... oh, I'll say, … I don't know how to define it, but truly, the doctor-patient relationship that, that is made of trust. When people rely wholly on the doctor, and therefore, coping with complications, it's annoying… but still, you have to do it a little… ! (Surgeon_03). |
| **Awareness of complications**  … when the liver wasn't good, when it wasn't working, I said, "this isn't possible, it's going to work, it will work, it will work" and it didn't ever work ... because, in fact, for a while, I was blind to my mistake … (Surgeon_05). |
| **Accountability because of intervention**  When there are complications, such as it won't heal, … in fact it's not that it won't heal but that the medication is keeping it from healing, and the complication is my fault, well, it's not my fault, it's the operation that I performed that is preventing her from healing well. (Resident_01).  We operate… that is, I mean, it's … we are very invasive, necessarily at some moment or another, even though we step back, we are directly responsible for what we do, I mean, if it goes badly, an operation always makes you feel guilty (Surgeon_03).  But the surgeon considers that it's HIS patient because he's the one who performed the operation and he's the one responsible for the patient and the consequences, and if it goes badly, it is in part his fault. (Surgeon_04). |
| **Pressure of the surgical ideal** |
| **Ideal position: Surgery is intended to cure.**  The aim is to take care of patients and cure them! (Surgeon_01).  It's a little soppy, but I think that's it's harder for the medical oncologist, who prescribes the chemo, while we do a procedure, the patient sees he has cancer, he leaves, he has no more cancer, all done … (Surgeon_06).  … that we have a viewpoint that is very, that is particular in surgery… we're in a process where we operate on people, so there's a process to cure them […]what we experience for a patient with a relapse, who has metastases, it's practically a failure (Surgeon_10). |
| **Institutional management of emotions and complications**  When you're very young, when you're an intern, well, then it's rubbish, you do stupid things, that's what it is. At the beginning it's training, then you are supposed to be trained. When you're no longer so young, you still do stupid things … but you can't do too many, because normally you should have some expertise that justifies that you're where you are now... […] which is a phase where…where you still feel evaluated by your peers, which is an important phase. Because precisely, in this phase, you don't have the right to make mistakes. And when you are older, you can do stupid things, because after all you have saved so many patients that you can do stupid things. […] for example X if he does something stupid in the OR, he will admit it, he'll say so … at least, I think so; and then no one will tell him he's stupid, because he's super-good, and everyone knows it (Surgon_05).  Even if there was a desire to do precisely this ....morbidity and mortality meetings to discuss all this, somewhere there is always some sort of … judgment […] you realize when you dissect the thing that there is always human error; so, often always a desire not to hurt someone, people always try to find a ... not quite human error, so to speak; but there is always a human error, there is always someone responsible (Surgeon_05).  … they told me, no you couldn't have done otherwise, blah blah, to please me, I think […] I'm the old man on the team, so the guys, my collaborators, I think they like me a lot and they,…they didn't lay into me, saying, but Sir, you shouldn't have done it like that! No, they said, ah but that's not how it's supposed to be done, and I said, I think you can do it differently, they said, but no, it's not possible etc… And then I discussed it with others as well, they said, no, well, there was another way to do it BEFORE, you understand what I mean, so that that doesn't happen… (Surgeon_12). |
