# Supplementary material for: Surgeons’ Emotional Experience of Their Everyday Practice - A Qualitative Study
Source: PLoS One. 2015 Nov 24;10(11):e0143763. doi: 10.1371/journal.pone.0143763 (PMC4657990; doi:10.1371/journal.pone.0143763)
Supplement: S2 Table — (DOC) [file pone.0143763.s002.doc]

**Table S1**. Strategies used by surgeons to manage the patients’ emotions during the information process.

| **Factor,**  Definition | **Exemplifying quote** |
| --- | --- |
| **Scientific knowledge** | |
| *Amount of risk*  Frequency and seriousness of possible complication. | … for each operation, the two or three most frequent complications, but we don't list all the complications (Resident_02).  If a rate is as low as 15%, I don't give it. I lie […]... Because they don't need to know the details like that, I don't think ….. Unless we have enormous responsibilities in relation to … society or something like that. […] It's not that they shouldn't know, but not everything (Surgeon_14). |
| *Availability of alternatives*  It is not necessary to communicate all risks if, finally, the patient has no other choice. | That depends, for the liver that we're operating on for tumors, cancer, if we leave it like that, they are going to die of it, so the question doesn't need to be asked. On the other hand, if the procedure is for something benign, and they must be monitored, for example, by scanner, every 3 months or every 6 months or forever, there are people who say, "no, I prefer that you operate, so that I won't have to do scans or monitoring, I would prefer you operate”. And then, you tell them, it's not nothing (easy?) to operate, there is this or that complication, and in the worse of cases, you could die. There, you can present it like that. (Surgeon_03). |
| **Singularity of the patient** | |
| *Psychological impact of information*  Surgeons tend to protect patients from the distress induced by the information | Eh, because you owe the information to the patient on condition that he can understand it, and that he won't kill himself when he leaves (Surgeon_15).  You can't… I think you should tell them more, but it's difficult… It's a little as if you said to a patient whose gall bladder you are taking out, you know you can die (Resident_01).  That is to say that it must be the surgeon's worry/problem, that any patient he operates on might die (Resident_02). |
| *Patients’ individual needs*  How surgeons evaluate patients’ preferences | Surgeon: Someone who must see, in this serious diagnosis, there must be someone who has all the information. Either it's the patient, if he wants to assume the burden, if he can, by himself… »  *Interviewer: How do you evaluate that?*  Surgeon: I don't know! Like this… Uh… a young guy, with a fairly young wife, he doesn't want to tell him everything, he wants to take it on himself … Uh, it’s often the man, I find. And for the older guy, it's more often the woman, who does not want her dependent husband to know everything… There we say everything! […] And you sense it, as you go along, you feel it … Sometimes there are people, you realize clearly that you mustn't tell them… (Surgeon_06).  For me, a good physician is the one who succeeds in sorting this out and who succeeds in being sufficiently human as well as being a good technician and also understands something of the psychology of the specific patient (Surgeon_03). |
| **External constraints (culture, society, institution)** | |
| *The surgeon*  Cultural attitude of both the individual surgeon and the medical system. | In France it's nonetheless a little more, it's still a little more paternalistic. We take care of the patient… We try to give him or her information, as they say, that's honest, clear and appropriate (Surgeon_04).  We are also there to use our knowledge and the knowledge in the literature to provide useful information, not to give him a list without weighting it -- is there a serious risk or a very low risk (Resident_02). |
| *Legal concerns*  Concerns about possible legal persecution for fault of information in case of complication | It's uh, … currently, finally I, ultimately, I don't give a damn about the medicolegal..., uh, to give you an example, it's the only thing you can attack a physician today for, it's a flaw in the information… Especially a hospital staff physician (Surgeon_08).  After, there's the legal information … You cannot perform pancreatic surgery without mentioning a fistula, you can't do liver surgery without mentioning a hemorrhage… Uh… it's surgery, without mentioning death… (Surgeon_06). |
