# Supplementary material for: Surgeons’ Emotional Experience of Their Everyday Practice - A Qualitative Study
Source: PLoS One. 2015 Nov 24;10(11):e0143763. doi: 10.1371/journal.pone.0143763 (PMC4657990; doi:10.1371/journal.pone.0143763)
Supplement: S3 Table — (DOC) [file pone.0143763.s003.doc]

**Table S2.** Domains of emotional impairment

| **Initial acknowledge and acceptance** |
| --- |
| When the liver wasn't good, when it wasn't working, I said, "this isn't possible, it's going to work, it will work, it will work" and it didn't ever work ... because, in fact, for a while, I was blind to my mistake… (Surgeon_05).  I can no longer stand, especially at my age, I can't stand complications… It has to be perfect, there must be nothing, it has to be impeccable … The patient must be in good condition, not need a transfusion, the operation must take place as I want, with no jokes, everyone has to be available. (Surgeon_12). |
| **Accountability for the error** |
| We operate… that is, I mean, it's … we are very invasive, necessarily at some moment or another, even though we step back, uh… we are directly responsible for what we do, I mean, if it goes badly, an operation always makes you feel guilty. I don't know a surgeon who, even when he's done well, doesn't feel guilty when a patient has a complication, and he tries to understand why! And if somehow it didn't result from a fault, or a … something during the procedure.. (Surgeon_03).  I've been angry at myself, even though retrospectively, completely objectively, I didn't find any cause for the complication (Surgeon_05). |
| **Difficulty in forgetting** |
| In 2011, of all the patients with liver and pancreatic surgery, and that is, after all, a few more than 200 patients, all together, so there was one death… Which is nothing, compared with what has been published, you know, it's practically zero mortality for very major surgery -- the pancreas and liver! So, that's good! But the patient who died that year, I still think about her, partly because I think that she was the only one who really shouldn't have died, you see! [..] Because she was 30 years old, she had two small kids, and she died! And she died of a complication… that we should have been able to avoid… We should have been able to avoid it… (Surgeon_12). |
| **Encounter with the family** |
| the dead patient ... I went to see the family, who I know well, since I've already operated on three family members for the same disease; it was a really awful experience, the next day I was ... It was horrible (Surgeon_12). |
